# Detection of Rare Beta Globin Gene Mutation [+22 5UTR(G>A)] in an Infant, Despite Prenatal Screening

**DOI:** 10.1155/2013/906292

**Published:** 2013-04-14

**Authors:** Mohammad Reza Mahdavi, Hosein Karami, Mohammad Taghi Akbari, Hosein Jalali, Payam Roshan

**Affiliations:** ^1^Thalassemia Research Center, Mazandaran University of Medical Sciences, Sari, Iran; ^2^Tarbiat Modares University, Tehran, Iran; ^3^Fajr Medical Laboratory, Sari, Iran

## Abstract

*Background*. Beta thalassemia is one of the most common hereditary disorders worldwide. In Iran, it is frequently reported from northern and southern provinces. In order to prevent child birth to be affected by this complication, prenatal screening and diagnosis are carried out nationwide. However, in some instances, this program is unable to identify rare mutations leading to thalassemia. *Case Presentation*. A married couple, who took part in prenatal screening and diagnosis, gave birth to a child who is affected by thalassemia major. After several molecular examinations, a rare mutation [+22 5UTR (G>A)] in compound heterozygote state along with a common mutation [codon 8 (-AA)] was found. *Conclusion*. This case study suggests that more advanced molecular evaluations must be integrated in prenatal screening programs to identify rare mutations and antenatal diagnosis of thalassemia cases.

## 1. Introduction

Beta thalassemia is one of the most prevalent autosomal received disorders in the world that has affected more than 150–200 million people from more than 60 countries around the world. About 18000 beta thalassemia major patients live in Iran, and it is estimated that the number of beta thalassemia carriers reaches to two million people, while most of them are in northern and southern provinces. Genetic counseling, identification of mutations responsible for the disease and carrier persons, and prenatal diagnosis are the best methods for the management of the disease and prevention of emergence of new cases in the community. All of these services are routinely available in Iran [1–4].

So far more than 200 different mutations on beta globin gene that lead to beta thalassemia disease have been identified. Molecular investigations on beta thalassemia patients in Iran resulted in finding 43 different beta globin gene mutations, responsible for the disease. These mutations have different frequencies in different provinces of Iran, with various ethnicities. In each province, some mutations are classified as common mutations, while others are known as rare mutations. In all provinces of Iran 13 mutations cover more than 70–90% of identified mutations among beta thalassemia patients that are classified as common mutations. In Mazandaran province (a northern province of Iran), IVS-II-1(G>A) mutation alone, with the prevalence of 61% among affected persons, is the most common mutation among beta thalassemia patients, and codon 30 (7.5%), codon 22 (6.2%), and codon 8 (-AA) (5.4%) mutations are other common mutations following IVS-II-1 (G>A) mutation [3, 5, 6].

+22 5UTR (G>A) mutation, which is one of the rare mutations causing beta thalassemia disorder, was first identified in Turkey. Unlike other beta thalassemia gene mutations, it was reported that in some instances hematological indices were less effective in individuals carrying +22 5UTR (G>A), and thus identification of beta thalassemia minor individuals carrying this mutation is more difficult than other carriers. It is suggested that this mutation causes inhibition of binding of mRNA to ribosome, and as a result, translation process cannot be done precisely [7]. In several provinces of Iran this mutation was reported. In Kurdish population of Iran this mutation was reported once in beta thalassemia patients with IVS-II-1 (G>A) mutation in compound heterozygote state, and in Qazvin province it was identified in a patient along with IVS 1/5 mutation [8, 9]. In Mazandaran province no beta thalassemia major patient has been previously reported carrying +22 5UTR (G>A) mutation.

Management of beta thalassemia has certain protocols. The patients need blood transfusion, and for controlling blood iron and prevention of iron overload, serum iron level should be constantly monitored via certain methods, including measurement of serum ferritin or transferrin receptor. The excessive blood iron needs to be removed from blood circulation by iron chelators. Routine medical examinations and periodic heart, liver, and spleen function tests are highly recommended in beta thalassemia patients.

## 2. Case Presentation

An 18-month old-male patient with Mazandarani ethnicity was referred to Fajr Laboratory Center, Sari, Mazandaran, for the identification of hemoglobin disorders. Following complete blood count (CBC) (Table 1), hemoglobin electrophoresis was carried out using Minicap capillary electrophoresis method (Sebia, France). Hematological indices and electrophoresis results were compatible with beta thalassemia pattern, while the parents were screened before pregnancy and the mother was diagnosed as a carrier (beta thalassemia minor) and the father was diagnosed to be normal. The specialist physician approved to proceed to pregnancy.

**Figure 1:**
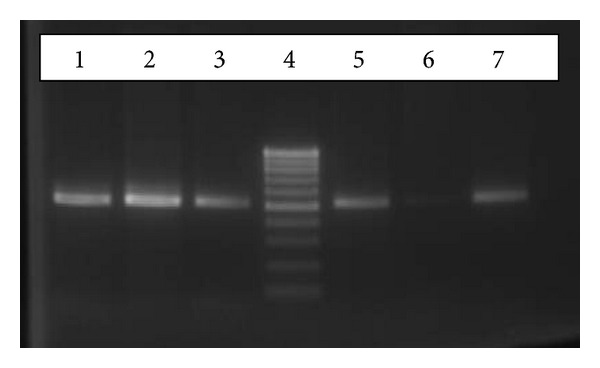
Gel agarose electrophoresis of ARMS-PCR product (522 bp fragment) for the detection of codon 8 (-AA) mutation. Patient sample: lanes 1 and 5, negative (wild type), control: lanes 2 and 6, positive control: lanes 3 and 7,and ladder marker 100 bp: lane 4. Lanes 1, 2, and 3 represent PCR products amplified with primers binding normal allele, and lanes 5, 6, and 7 represent PCR products amplified with primers binding mutant allele.

In order to detect mutations on beta thalassemia gene, at first five common beta thalassemia mutations were studied applying amplification refractory mutation (ARMS)-PCR technique. The result showed that the patient has codon 8 (-AA) mutation on heterozygote state, but another mutation causing beta thalassemia disorder remained unknown (Figure 1). Then, for the identification of another mutation reverse hybridization assay followed by multiplex PCR that simultaneously detected 22 mutations was used. The result confirmed heterozygosity for codon 8 (-AA) mutation, while second mutation still remained unidentified.

**Figure 2:**
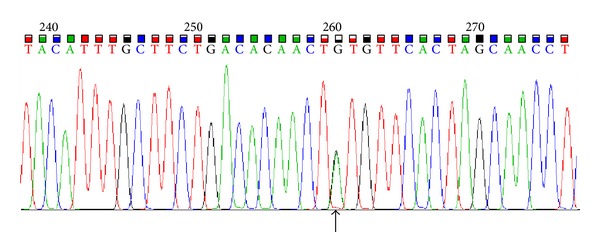
sequencing of beta globin gene showing location of +22 5UTR (G>A) mutation on beta globin gene.

Finally, for the detection of second mutation involved in manifestation of beta thalassemia disease we used DNA sequencing of beta globin gene using ABI377 instrument (Applied Biosystems, Foster City, CA, USA) after amplification of the gene with specific primers. Result of the sequencing showed two mutations for the patient: codon 8 (-AA) and +22 5UTR (G>A) in compound heterozygote state (Figure 2). The patient was also screened for 22 alpha globin gene mutations using PCR reverse hybridization assay technique and no mutation was found. Treatment of the patient was started before full identification of mutations, and after finding the mutations, the treatment has been continued with higher precision.

## 3. Discussion

In order to identify beta globin gene mutations, several studies have been carried out since 1980s, and after invention of PCR technique, the rate of finding new mutations significantly increased. So far more than 200 different mutations on beta globin gene—mostly point mutations, small deletions, or addition of few nucleotides—have been identified. Unlike alpha globin gene, large deletions are rarely reported in beta globin gene. Prevalence rate of mutation in different races and ethnic groups is varied, and in each geographical area some mutations are more common than the others. For example in Sadina Arabia and Italy, 95.7% of beta thalassemia patients have codon 39 C>T mutation [10]. Nevertheless, always there are some rare mutations among beta thalassemia patients, and identification of all rare mutation in each region can help improve screening protocols of the carriers and prevent affected child birth.

In 2007, Derakhshandeh-Peykar et al. investigated the presence of beta globin gene mutations among 394 beta thalassemia carriers in northern provinces of Iran using ARMS-PCR and DNA sequencing methods. In that study 19 different mutations were identified. IVS-II-1 (G>A) mutation had prevalence rate of 51.6% among all discovered mutations, and none of the cases had +22 5UTR (G>A) mutation [3].

In another study in 2011, Akhavan-Niaki et al. screened for beta globin gene mutations among 1635 beta thalassemia minor individuals. 998 of the cases (61%) had IVS-II-1 (G>A) mutation and +22UTR (G>A) mutation was found in only 2 cases (0.1%) [6]. As mentioned before, in our study this mutation was found with codon 8 (-AA) mutation in compound heterozygote state.

+22 5UTR (G>A) mutation, which is located on 5′ untranslated region of beta globin gene, is a rare mutation, and in Mazandaran no beta thalassemia patient with this mutation was previously reported. This mutation has been previously reported from two other provinces of Iran. It was in compound heterozygote form with other mutations: in Kurdistan province with IVS-II-1 (G>A) mutation and in Qazvin province with IVS-1/6 mutation [8, 9].

This study reported a beta thalassemia major patient with +22 5UTR (G>A) mutation in Mazandaran province, for the first time. Finding this case shows that there are some rare mutations that can be missed during screening program of beta thalassemia carriers, and it is recommended for this mutation to be considered in screening protocols for beta thalassemia carriers in Mazandaran.

## Figures and Tables

**Table 1:** Hematological indices of the patient and his parents.

	Gender	Age (y)	RBC (×10^6^/*μ*L)	Hb (g/dL)	Hct (%)	MCV (fL)	MCH (pg)	MCHC (g/dL)	Hb-A (%)	Hb-A2 (%)	Hb-F (%)
Case	M	1/5	4/09	7/3	23/1	56/5	17/8	31/6	35/2	3/2	61/6
Father	M	30	5/78	12/4	42/8	67/7	21/5	31/7	96/8	2/6	0/6
Mother	F	26	5/3	9/8	32/9	62/1	18/5	29/8	93/8	5/7	0/5
